# Classic Kaposi Sarcoma: to treat or not to treat?

**DOI:** 10.1186/s13104-015-1076-1

**Published:** 2015-04-10

**Authors:** Bruno Vincenzi, Loretta D’Onofrio, Anna Maria Frezza, Rosario Francesco Grasso, Valentina Fausti, Daniele Santini, Angelo Paolo Dei Tos, Giuseppe Tonini

**Affiliations:** Department of Medical Oncology, University Campus Bio-Medico, via Alvaro del Portillo 21, Rome, Italy; Department of Radiology, University Campus Bio-Medico, via Alvaro del Portillo 21, Rome, Italy; Department of Pathology, General Hospital, P.zza Ospedale 1, Treviso, Italy

**Keywords:** Classic Kaposi sarcoma, Spontaneous regression

## Abstract

**Background:**

Classic Kaposi Sarcoma (KS) is vascular sarcoma, known to be more common in Mediterranean elderly men and characterized by an indolent clinical behavior. To our knowledge, this is the first evidence in literature, describing a spontaneous partial regression in a non-HIV, non-iatrogenic KS.

**Case presentation:**

A 68-years old woman, presenting with weight loss and respiratory symptoms, was diagnosed with a classic KS involving lungs and mediastinal lymph nodes. No skin or mucosal lesions were identified, HIV positivity was ruled out. Due to patient’s choice, she was kept under surveillance with 3-monthly thorax-abdomen-pelvis computed tomography scan (TAP CT). A first reassessment proved progressive disease (PD) associated with symptoms worsening. A new TAP CT, performed at 5 months from the diagnosis, showed stable disease (SD), with a minor reduction in size of mediastinal lymphadenopathies. A further reassessment, performed 5 months later, resulted in a partial response (PR) despite the absence of any medical treatment. Up to date, the disease is in remission, patient is asymptomatic and still on surveillance.

**Conclusion:**

Given the possible indolent behaviour of KS, we believe that close surveillance can represent a valuable approach in selected cases.

## Background

KS is a locally aggressive endothelial tumour belonging to the family of vascular sarcoma. According to aetiology and epidemiology, four variants have been described: classic KS, usually affecting Mediterranean elderly men; endemic African KS, common in middle-aged adults and children in Equatorial Africa; iatrogenic KS, usually occurring in solid organ transplant recipients but also in patients receiving immunosuppressive treatment (i.e. corticosteroid) for a long time; acquired immunodeficiency syndrome associated KS, the most aggressive variant, affecting human immunodeficiency virus (HIV) positive patients. KS is invariably associated with human herpes virus type 8 (HHV-8), which seems to play a key role in KS pathogenesis [1]. KS typically presents with mucocutaneous lesions, mostly affecting lower extremities, face, trunk, genitalia and oropharyngeal mucosa, but it can also involve lymph nodes and visceral organs, including the respiratory and gastrointestinal tracts [2]. Clinical behaviour is often indolent, especially in the classic variant.

Pulmonary involvement is common in critically immunodeficient patients, occurring approximately in 45% of those with cutaneous AIDS-related KS with previous or concomitant mucocutaneous lesions. Lung metastases from sporadic KS are rare, especially in female and pediatric series with a distinct male predominance [3]. Treatment options for non-HIV forms include chemotherapy (liposomal doxorubicin, vinblastine, taxanes) [4], immunotherapy (interferon alpha, interleukin-12) and anti-HHV8 therapy [5]. Close surveillance can also be a possibility in selected cases.

## Case presentation

**Figure 1:**
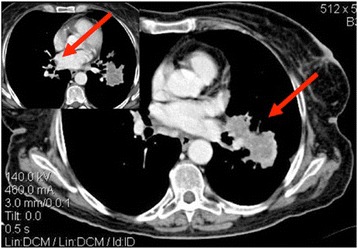
Baseline thorax-abdomen-pelvis computed tomography, showing a large mass in the left lower lobe, multiple bilateral pulmonary nodules and mediastinal adenophaties.

**Figure 2:**
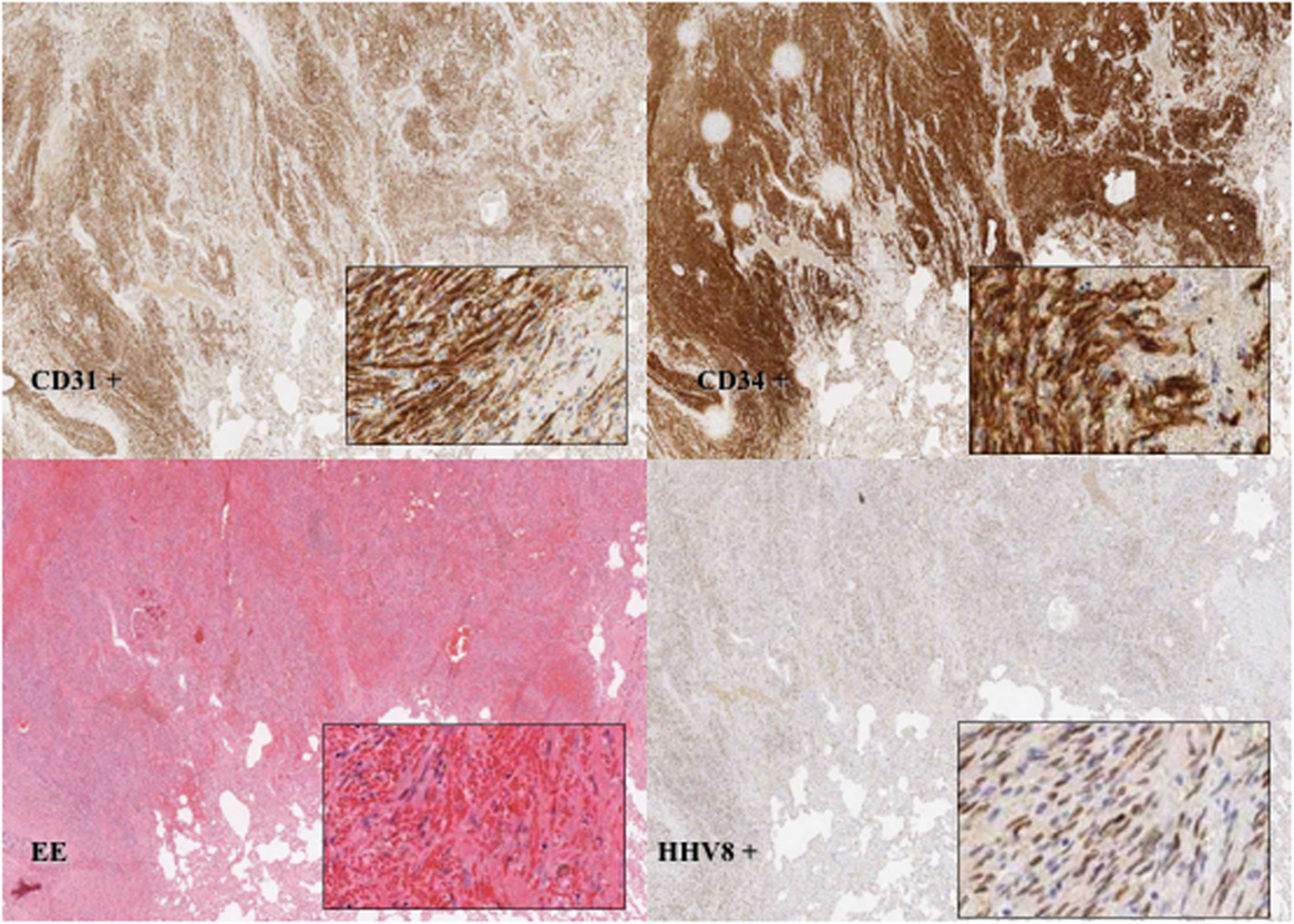
Histology and immunohistochemistry (CD31, CD34 and HHV-8).

A 68-years old woman, with no previous medical history, presented with severe weight loss, shortness of breath and cough (ECOG PS: 2). A chest x-ray followed by a thorax-abdomen-pelvis computed tomography (TAP CT) showed the presence of a 53 mm large mass in the left lower lobe (LLL) with pericardial involvment and a 30 × 18 mm large lesion with similar morphological features in the upper lobe of the left lung. Multiple bilateral lung nodules and a 26 mm large carinal lymphadenopathy were also reported (Figure 1). Bronchoscopic and CT-guided biopsies were attempted without success, ending up with a surgical exploration and complete removal of one lung nodule. Pathology proved a malignant stromal tumor with endothelial phenotype, consistent with a pulmonary localization of KS. Immunohistochemistry resulted positive for CD31, CD 34 and HHV-8; Ki67 10% (Figure 2). Physical examination ruled out any suspicious skin lesions, while upper gastrointestinal endoscopy and colonoscopy excluded mucosal localizations. Anti-HIV antibody and HIV antigen tests were both negative.

**Figure 3:**
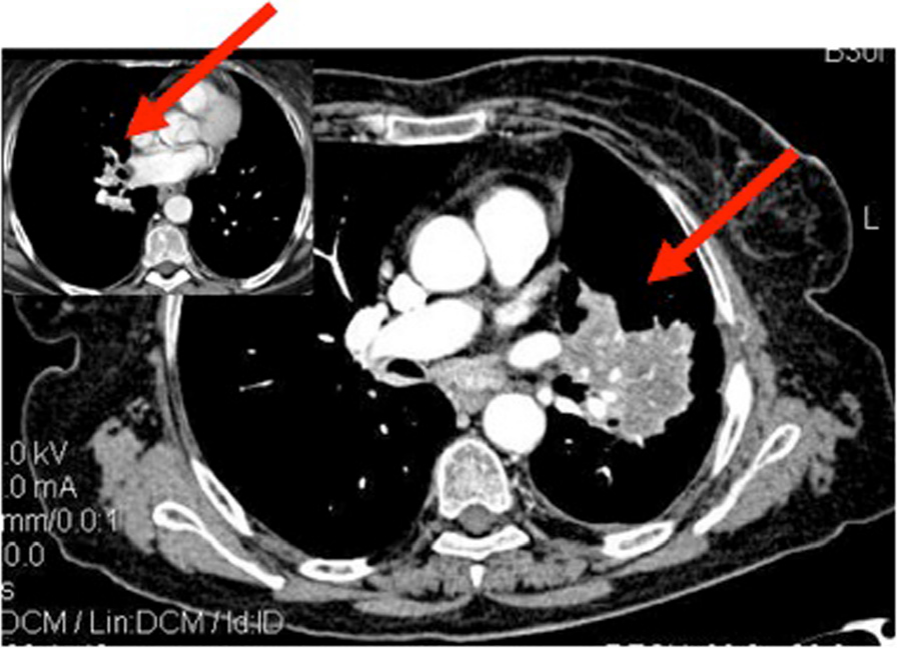
Thorax-abdomen-pelvis computed tomography two months after diagnosis, showing progressive disease.

**Figure 4:**
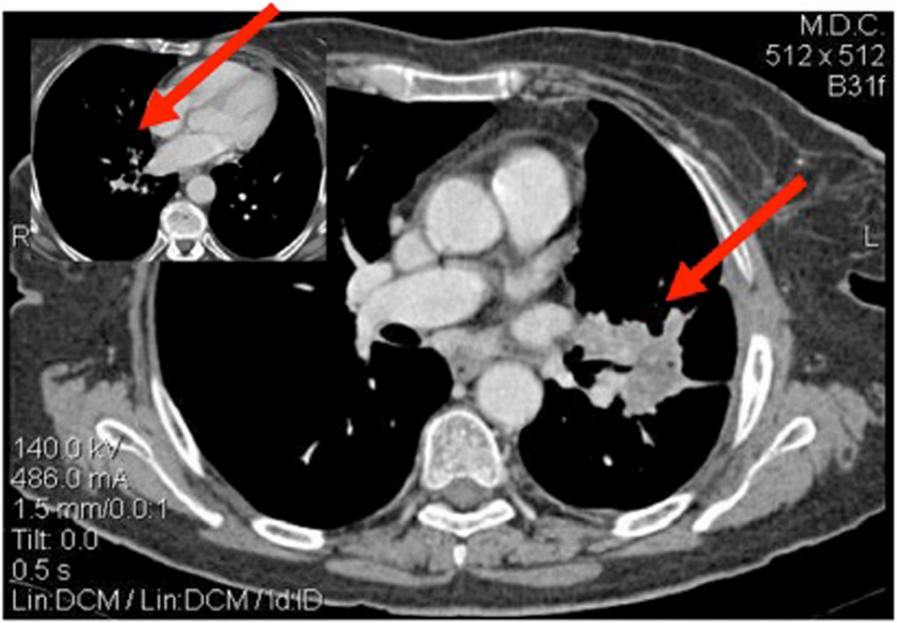
Thorax-abdomen-pelvis computed tomography two months after diagnosis, showing partial response.

Given patient’s refusal of any medical treatment, she was started on a surveillance program. The first reassessment proved PD according to RECIST 1.1 criteria, with the appearance of a new nodule in the right mid lobe (RML), an increase in size of the known lesion in the LLL (60 mm) and a stability of the carinal lymphnodes (Figure 3). Despite the persistence of symptoms, due to patient’s choice, no treatment was started. A new radiological assessment, performed after 5 months from diagnosis, showed a minor reduction in size of the lymphadenopathies and an almost complete resolution of the nodule in the RML. Given radiological evidence of disease regression and improvement in symptoms, the patient underwent a further TAP CT five months later, showing PR, with a reduction in number of lung nodules and reduction in size on both the lesion in the LLL and carinal lymphnodes (Figure 4). Up to date, at nineteen months from diagnosis, disease is still in remission with stable pulmonary nodules and no evidence of systemic spread, in absence of any medical treatment. The patient is still on surveillance through three-monthly clinical assessment and six-monthly TAP CT, asymptomatic, ECOG PS: 0.

## Conclusion

Spontaneous regression of KS has been previously described in the iatrogenic type. Interruption of corticosteroids [6], systemic immunosuppressive drugs [7,8], and ACE-inhibitors [9] can lead to a spontaneous self-healing of KS skin lesions. In AIDS-related subtype, disease regression usually results from intensive anti-retroviral therapy [10,11]. To our knowledge, this is the first evidence in literature, describing a spontaneous partial regression in non-HIV, non-iatrogenic Kaposi sarcoma. In Kondo’s paper [12], multiple skin lesion histologically suspected for sporadic Kaposi sarcoma, disappeared after the discontinuation of a prolonged steroid therapy administered for chronic respiratory failure. In the present case report, no trigger mechanisms leading to the development of this disease were identified, nor removable stimuli to malignant proliferation. There are no data in literature providing evidence for possible biological mechanism underlying this spontaneous regression of disease. Conversely, the presence of herpes virus infection and any concomitant changes in the balance of immune system are well recognized factors promoting the development of this cancer [13-16]. Therefore, we might assume that a fully competent immune system, in absence of the impairment induced by chemotherapy administration, could have played a role in the observed disease regression. However, future studies are needed to verify the reliability of this hypothesis and, despite this interesting observation, more data are needed to support close surveillance as a management strategy in this rare disease.

## Consent

Written informed consent was obtained from the patient for publication of this Case Report and any accompanying images. A copy of the written consent is available for review by the Editor-in-Chief of this journal.

## Abbreviation

KS: Kaposi sarcoma
LLL: Left lower lobe
PD: Progressive disease according to RECIST 1.1 criteria
PR: Partial response according to RECIST 1.1 criteria
RML: Right mid lobe
SD: Stable disease according to RECIST 1.1 criteria
TAP TC: Thorax-abdomen-pelvis computed tomography scan
